# Video-based heart rate monitoring across a range of skin pigmentations during an acute hypoxic challenge

**DOI:** 10.1007/s10877-017-0076-1

**Published:** 2017-11-09

**Authors:** Paul S. Addison, Dominique Jacquel, David M. H. Foo, Ulf R. Borg

**Affiliations:** 1 Medtronic, Video Biosignals Group, Patient Monitoring, Technopole Centre, Edinburgh, EH26 0PJ UK; 2 Medtronic, Medical Affairs, Patient Monitoring, Boulder, CO USA

**Keywords:** Video monitoring, Heart rate, Skin pigmentation

## Abstract

The robust monitoring of heart rate from the video-photoplethysmogram (video-PPG) during challenging conditions requires new analysis techniques. The work reported here extends current research in this area by applying a motion tolerant algorithm to extract high quality video-PPGs from a cohort of subjects undergoing marked heart rate changes during a hypoxic challenge, and exhibiting a full range of skin pigmentation types. High uptimes in reported video-based heart rate (HR_vid_) were targeted, while retaining high accuracy in the results. Ten healthy volunteers were studied during a double desaturation hypoxic challenge. Video-PPGs were generated from the acquired video image stream and processed to generate heart rate. HR_vid_ was compared to the pulse rate posted by a reference pulse oximeter device (HR_p_). Agreement between video-based heart rate and that provided by the pulse oximeter was as follows: Bias = − 0.21 bpm, RMSD = 2.15 bpm, least squares fit gradient = 1.00 (Pearson R = 0.99, p < 0.0001), with a 98.78% reporting uptime. The difference between the HR_vid_ and HR_p_ exceeded 5 and 10 bpm, for 3.59 and 0.35% of the reporting time respectively, and at no point did these differences exceed 25 bpm. Excellent agreement was found between the HR_vid_ and HR_p_ in a study covering the whole range of skin pigmentation types (Fitzpatrick scales I–VI), using standard room lighting and with moderate subject motion. Although promising, further work should include a larger cohort with multiple subjects per Fitzpatrick class combined with a more rigorous motion and lighting protocol.

## Introduction

There is a major effort across patient monitoring to develop wireless solutions: whether to allow for untethering of the patient in the home environment or the de-cluttering of cables in-hospital. This can be achieved either through a wireless transmission technology where a probe (ECG, SpO_2_, rSO_2_, EEG, etc.) is still attached to the patient but the information is transmitted wirelessly or, alternatively, it may be carried out through a non-contact methodology where essentially the probe is at a distance from the patient (e.g., in the study described here, a video camera). The determination of physiological parameters from video image streams has attracted significant attention in recent years, with a variety of signal processing and hardware approaches employed [1]. A recent editorial by Thiele [2] stated that the “potential implications [of these technologies] are enormous” and that it may open a window “into the development of technology that may one day change our lives in ways we cannot imagine.” One area which might benefit greatly is the Neonatal Intensive Care Unit (NICU) setting, where existing methods for monitoring heart rate, respiratory rate and oxygen saturation for infants require the use of adhesive electrodes or sensors. These can damage the fragile skin of pre-term infants, and cause stress and pain [3, 4]. In their comprehensive review, Sun and Thakor [5] covered the use of non-contact video-based technologies in telemedicine and personal healthcare stating that such technologies would seem “to be a promising solution to enable elderly people to monitor their health conditions on a daily basis independently.” Zhao et al. [6] also proposed its use for telemedicine, in particular the remote measurement of heart rate and respiration rate using ambient light during the day and IR illumination at night. More recently, Li et al. [7] suggested its use for the monitoring of heart rate during sleep using IR illumination.

The acquired signal—the video photoplethysmogram (video-PPG)—is highly susceptible to three main confounders: motion artefact, lighting levels (both low levels and dynamic variations) and skin pigmentation. A variety of powerful signal processing methods has been proposed to determine heart rate from the video-PPG. These include frequency-based methods such as spectral peak tracking [8], and Fourier-based amplitude spectrum and phase spectrum adaptive filter for motion compensation [9]; wavelet transform time–frequency methods including the weighted transform component method [10], a dual tree complex wavelet transform method [11] and a running wavelet archetyping (RWA) method [12]; independent component analysis (ICA) methods such as the temporal constrained ICA and adaptive filter method [13], an assessment of three different ICA methods [14] and a comparison of independent component analysis, principle component analysis (PCA) and cross-correlation [15].

The effect of skin pigmentation on the video-PPG has been investigated by a number of researchers. The variation in skin tone may be stratified according to the Fitzpatrick scale [16], which varies from I to VI: the lightest to darkest skin tones respectively. Bousefsaf et al. [17] included a range of skin tones, from Fitzpatrick I to IV in their cohort of 12 volunteers on whose data they performed wavelet analysis of the video-PPG to derive instantaneous heart rate. They did not, however, stratify their results relative to skin tone. In describing the challenging conditions met during their neonatal study, Aarts et al. [3] noted the relatively small signal strength they obtained from a neonate with skin type V. However, in a detailed examination of the effect of skin pigmentation the same group, [18] noted a distinct downward trend in signal-to-noise ratio (SNR) of the extracted video-PPGs as the Fitzpatrick scale increased from I through to VI, with a factor of 2 difference in SNR occurring between type I and VI. Subsequently, Wang et al. [19] tested 8 algorithms for extracting the video-PPG using data from a 15 healthy subject cohort where they split the skin tones into three subgroups: [I and II], [III], [IV and V]. They found that skin types IV and V produced the worst SNR values for all eight algorithms. A distinct reduction in SNR with increasingly darker skin tone can be observed in the plotted results of Shao et al. [20], who examined the ballistocardiographic and photoplethysmographic signals simultaneously from a cohort of 23 subjects. The reduction in SNR with skin pigmentation scale was similar to that found by de Haan and Jeanne [18] described above, although across a smaller rage of Fitzpatrick scales (II–V).

The work reported here extends current research in this area by studying a cohort of healthy volunteers exhibiting distinct heart rate changes due to being subjected to a rigorous, protocolized hypoxic challenge and incorporating a full range of skin pigmentation types according to the Fitzpatrick scale (scales I–VI). The algorithm used to determine heart rate was constructed in order to extract valid video-PPG signal across the full range of skin types in relatively low room lighting condition. In addition, a degree of motion tolerance was desired to address the natural movement of the subjects during the challenge. In this way we strove to achieve high uptimes (the percentage of time that the video-PPG can be calculated while a valid reference signal is available) while retaining accuracy in the measurements.

## Methods

### Clinical study

The data was collected opportunistically during a non-related study to measure the effect of various interventions on the reported values of another monitoring device. Part of this parallel study protocol involved two desaturation events conducted in close succession, and data from these events were collected for the video research work. The study also involved the acquisition of reference pulse oximetry pulse rate data (Nellcor N600x™, Medtronic, Boulder, CO). Approval was given for the use of video monitoring and no other alteration to the existing protocol was made.

**Fig. 1:**
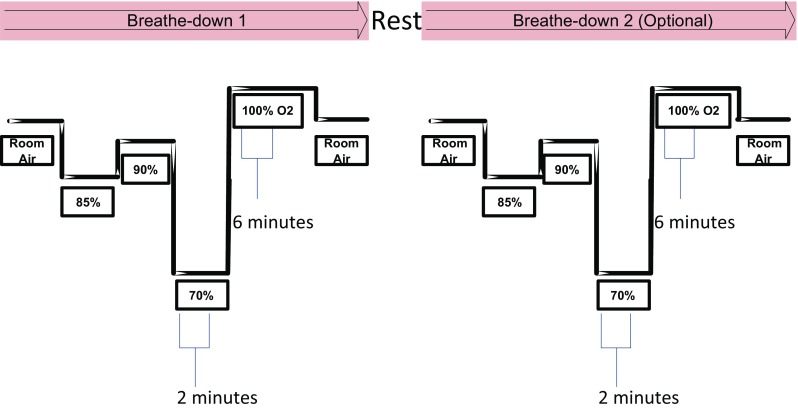
Desaturation protocol

**Fig. 2:**
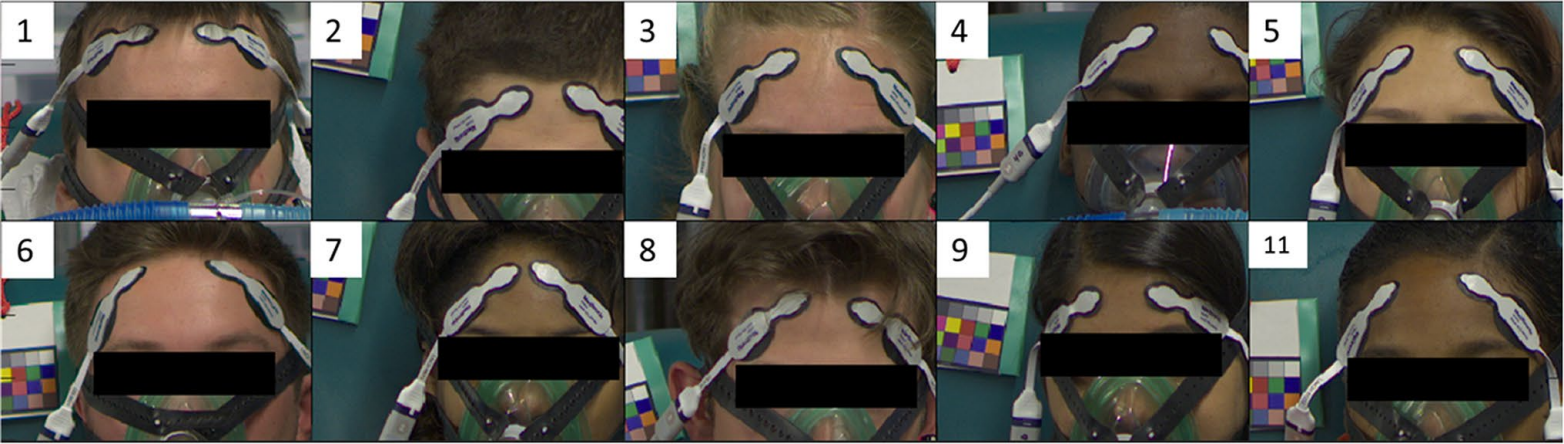
De-identified video stills of the ten subjects who completed the trial

Eleven healthy volunteers participated in the study. Subjects provided an institutional review board (IRB) approved informed consent covering the essential information stated in the protocol, as required elements according to 21 CFR 50.25 (27) for a non-significant risk medical device investigation. The subjects were fitted with a face mask in order to adjust the FiO_2_ using a mixture of nitrogen and oxygen and induce desaturation. Each subject underwent two discrete episodes of hypoxia. The sequence of targeted oxygen saturation levels is highlighted in Fig. 1. One subject could not tolerate the hypoxic challenge and the procedure was halted for this subject early during the first desaturation. The limited data from this subject was not used in the analysis. Video signals from 20 hypoxic episodes were therefore collected from the remaining 10 subjects who completed the protocol. The subjects had a mean age of 25.8 (standard deviation, SD 4.5) years and mean body mass index (BMI) of 24.07 (SD 1.55) and ranged across the whole Fitzpatrick skin tone scale of I–VI. The subject characteristics leading to the Fitzpatrick classifications are provided in Table 1. De-identified screenshots of the subjects are shown in Fig. 2.

**Table 1:** Demographic information including pigmentation characteristics and Fitzpatrick Scale Classification (note that subject 10 is missing as she did not complete the test, resulting in a highly incomplete data set.)

Subject ID	Gender	Age (years)	Weight (kg)	Height (cm)	BMI	Skin tone	Eye color	Hair color	Fitzpatrick classification
1	Male	26	89.9	193.0	24.1	Fair	Light brown	Brown	II
2	Male	29	70.4	167.6	25.0	Olive	Light brown	Dark brown	III
3	Female	28	65.8	165.1	24.2	Fair	Light green	Blond	I
4	Male	29	86.3	177.8	27.3	Very dark brown	Dark brown/black	Black	VI
5	Female	23	65.4	165.1	24.0	Olive	Hazel	Brown	III
6	Male	22	68.1	177.8	21.5	Fair	Light brown	Light brown	II
7	Female	22	59.0	157.5	23.8	Medium brown	Dark brown/black	Dark brown/black	IV
8	Male	24	79.5	188.0	22.5	Fair	light green	Light brown	I
9	Female	20	68.1	165.1	25.0	Medium brown	Dark brown/black	Black	IV
11	Female	35	63.6	165.1	23.3	Dark brown	Dark brown/black	Black	V

### Data acquisition and processing

#### Hardware

The video image stream was captured using a scientific camera (Basler acA1920-155uc with Nikon AF-S NIKKOR 35 mm 1:1.8G lens) at a frame rate of 70 fps. Video footage was acquired during each of the two desaturation events. The camera was placed on a tripod at approximately 2 m from the subject’s head and the data streamed to a laptop via a USB cable. Subject illumination was via fluorescent room lights on the ceiling, approximately 2 m above the subject’s head. Light intensity at head level was 250 lx measured using a DT-1309 light meter (CEM, Shenzhen, China).

The production of a robust heart rate requires two main steps: a biosignal extraction algorithm to produce the video-PPG from the video image stream and a vital sign processing algorithm which processes the video-PPG in order to determine the heart rate. These are discussed detail as follows:

#### Biosignal extraction

**Fig. 3:**
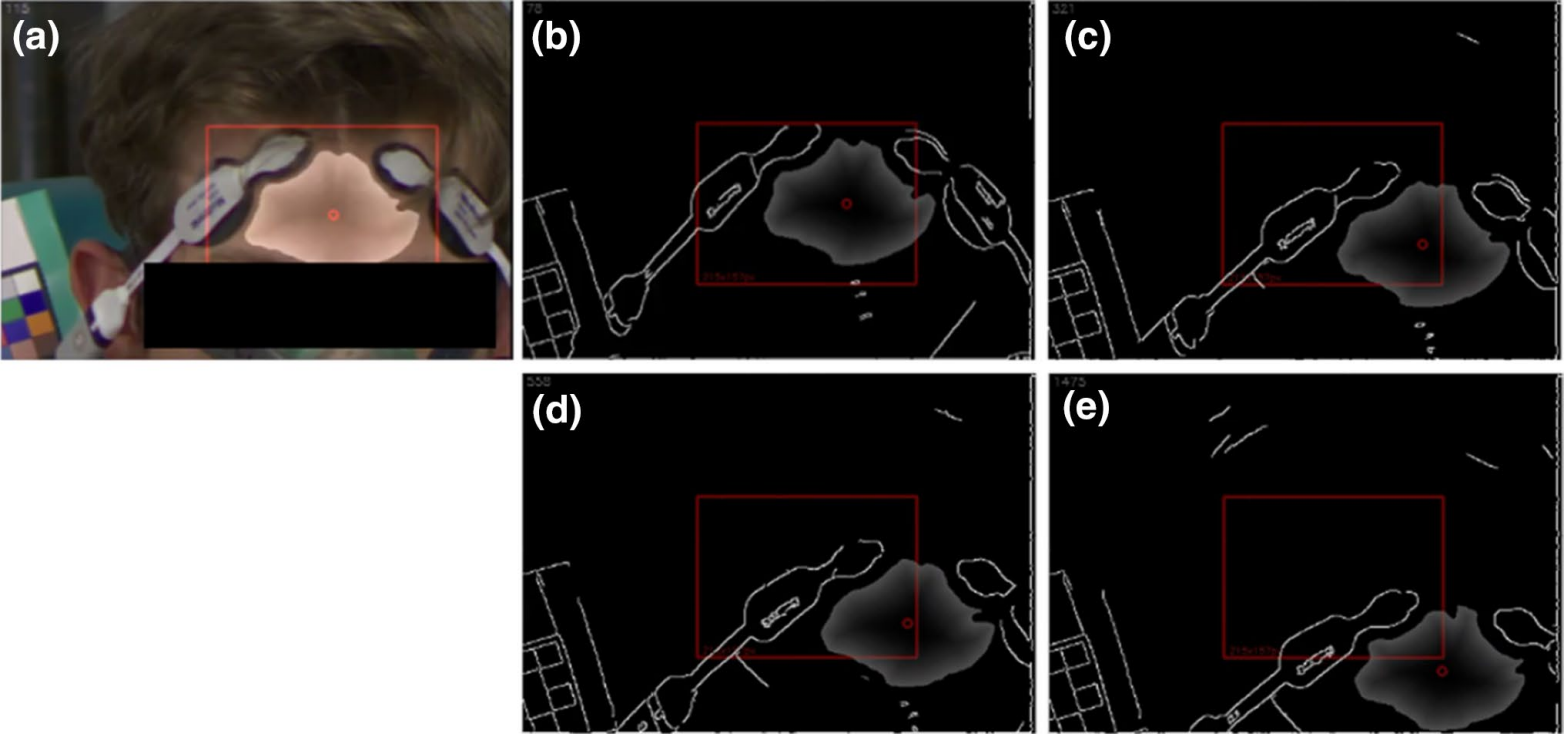
Example sequence of Images during a desaturation. **a** Original video still of one of the healthy subjects at the beginning of a desaturation sequence. **b**–**e** Four line drawing stills from the video over time showing the extent of movement in the Frame. **b** Is the line drawing version of **a**

**Fig. 4:**
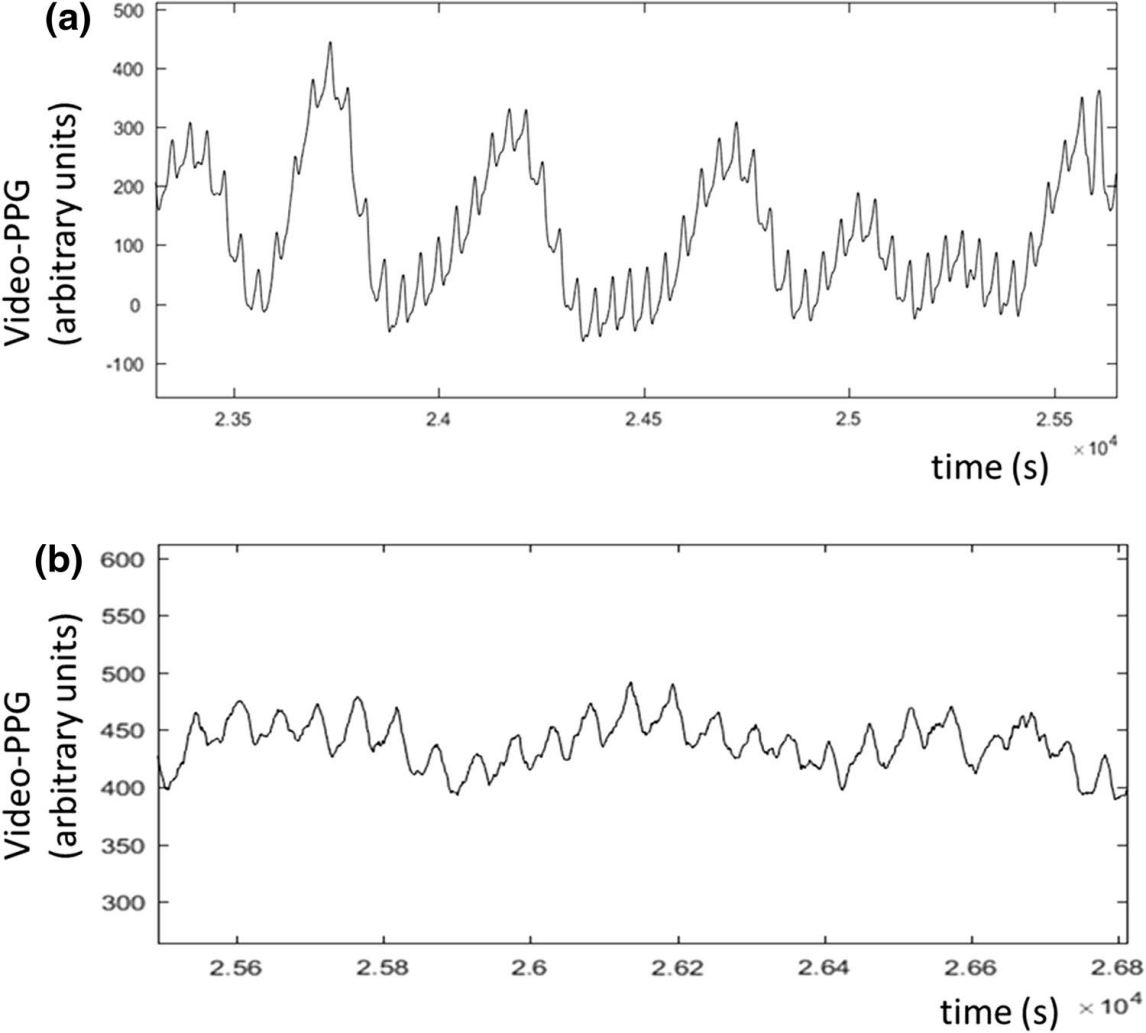
Two examples of the aggregated video-PPG signals generated using the camera system and flood field tracking method. **a** Relatively clean cardiac pulses superimposed on large scale respiratory motion. **b** Less well defined pulses superimposed on a lower amplitude respiratory waveform

The video-PPG signal was generated from a region of interest (ROI) on the image selected using a flood-field algorithm. This comprised identifying a seed point on the forehead, setting lower and higher tolerances for skin color around the seed point representing the maximum allowable relative changes between adjacent pixels, and performing a flood fill operation for each frame which recursively aggregated all adjacent pixels within the pre-set tolerance, starting from the seed point. The mean pixel modulations across the field were then used to construct the video-PPG. Figure 3 contains a sequence of images showing the flood fill field being tracked during motion. Two examples of the video-PPG captured during the study are presented in Fig. 4 showing cardiac pulses superimposed on respiratory modulations.

#### Vital sign processing

Once generated, the video-PPG requires further processing to extract the physiological parameter of interest: here the heart rate. This comprised the processing of the green channel using a fast Fourier transform (FFT) applied over a sliding temporal window of length 18 s. The video heart rate (HR_vid_) was computed automatically from the resulting frequency spectra by an algorithm which determined the physiologically relevant local peaks in the spectrum: this was defined as the dominant peak in the spectrum in a range between 0.5 and 4 Hz corresponding to heart rates between 30 and 240 beats per minute.

A Nellcor™ sensor (Max-A, with Oximax™ technology, Medtronic, Boulder, CO) was attached to the subject’s finger and provided a reference pulse rate (HR_p_). The video derived heart rate was then aligned in time with the reference rate to allow a comparative analysis of the two signals as described in the next section.

### Data analysis

Bias and accuracy statistics were calculated to compare the video-derived heart rate with the pulse oximeter rate. These are, respectively, the mean difference and the root mean squared difference between the test and reference values. That is,

1 $$bias=\frac{{\mathop \sum \nolimits_{{i=1}}^{N} \left( {H{R_{vid}}(i) - H{R_p}(i)} \right)}}{N}$$

and

2 $$accuracy=\sqrt {\frac{{\mathop \sum \nolimits_{{i=1}}^{N} {{\left( {H{R_{vid}}(i) - H{R_p}(i)} \right)}^2}}}{N}}$$

The latter expression is root mean square deviation (RMSD) and represents a combination of the systematic and random components of the error.

Least-squares linear regression was performed to obtain the line of best fit between the video and reference parameters from which the gradient, intercept, Pearson correlation coefficient *R* and associated *p* values were computed. In this work p < 0.05 was considered statistically significant.

A Bland–Altman analysis of the data was also performed using the method of [21] which compensates for within-subject longitudinal correlation in the data. SD of the bias and corresponding limits of agreement were calculated using this methodology.

Outlier statistics E5, E10 and E25 were calculated: these are defined as the percentage of the time that the absolute difference between the video heart rate and pulse oximeter reference exceeded 5, 10 and 25 bpm respectively and provide an indication of the algorithm’s susceptibility to reporting markedly erroneous results.

An uptime was determined, defined as the percentage of time that each parameter could be calculated when there was a valid reference value posted by the pulse oximeter.

The described signal and image processing were performed using in-house C++ code. Statistical analysis was carried out in Matlab, version R2016b.

## Results

**Fig. 5:**
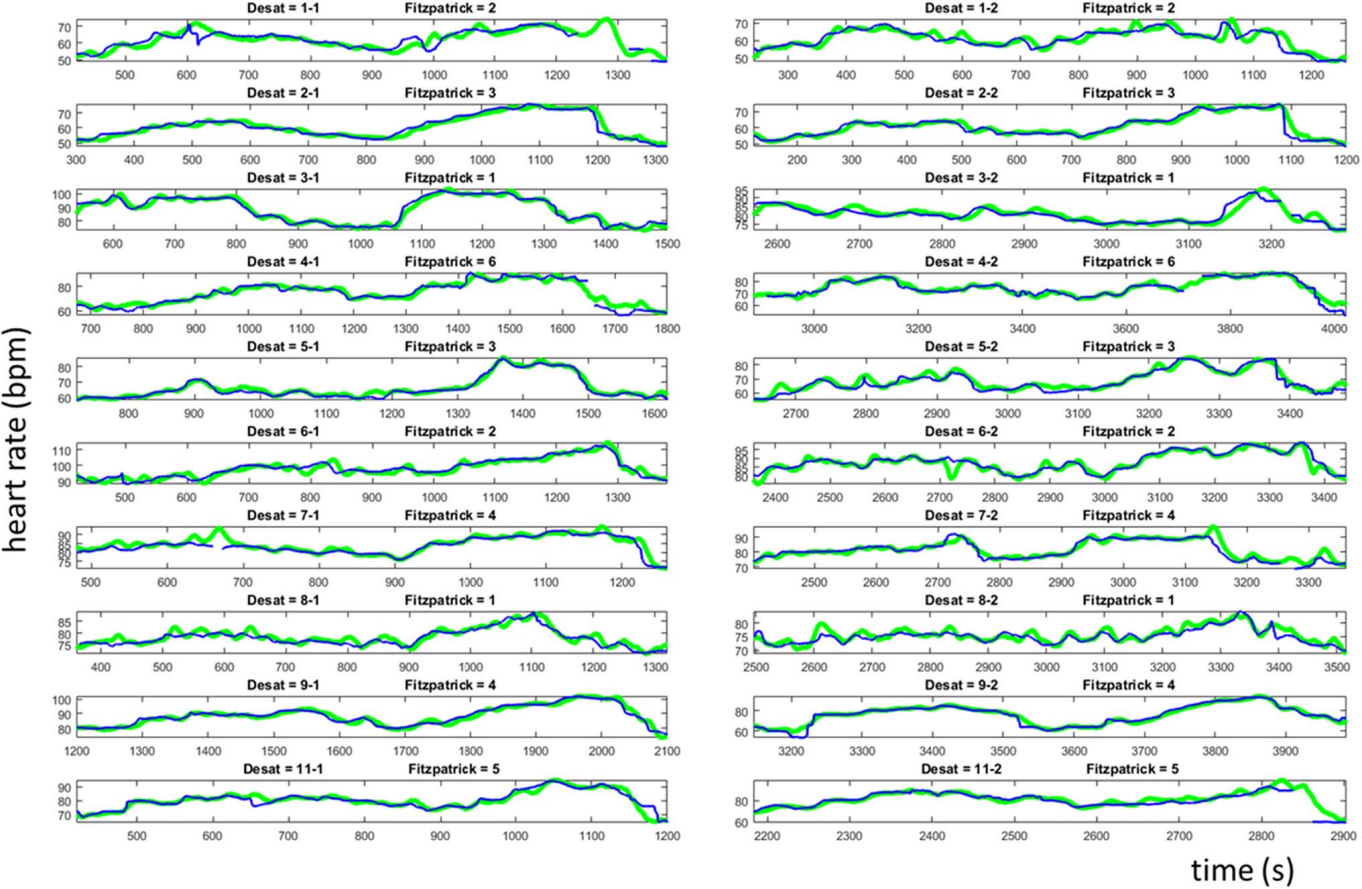
Time series of video and pulse oximeter heart rates for the 20 desaturations [10 subjects (rows) and 2 desaturations per subject (columns)]. (HR_vid_ = blue. HR_p_ = green). The left hand signals correspond to the first desaturation and the right hand signals to the second desaturation

**Fig. 6:**
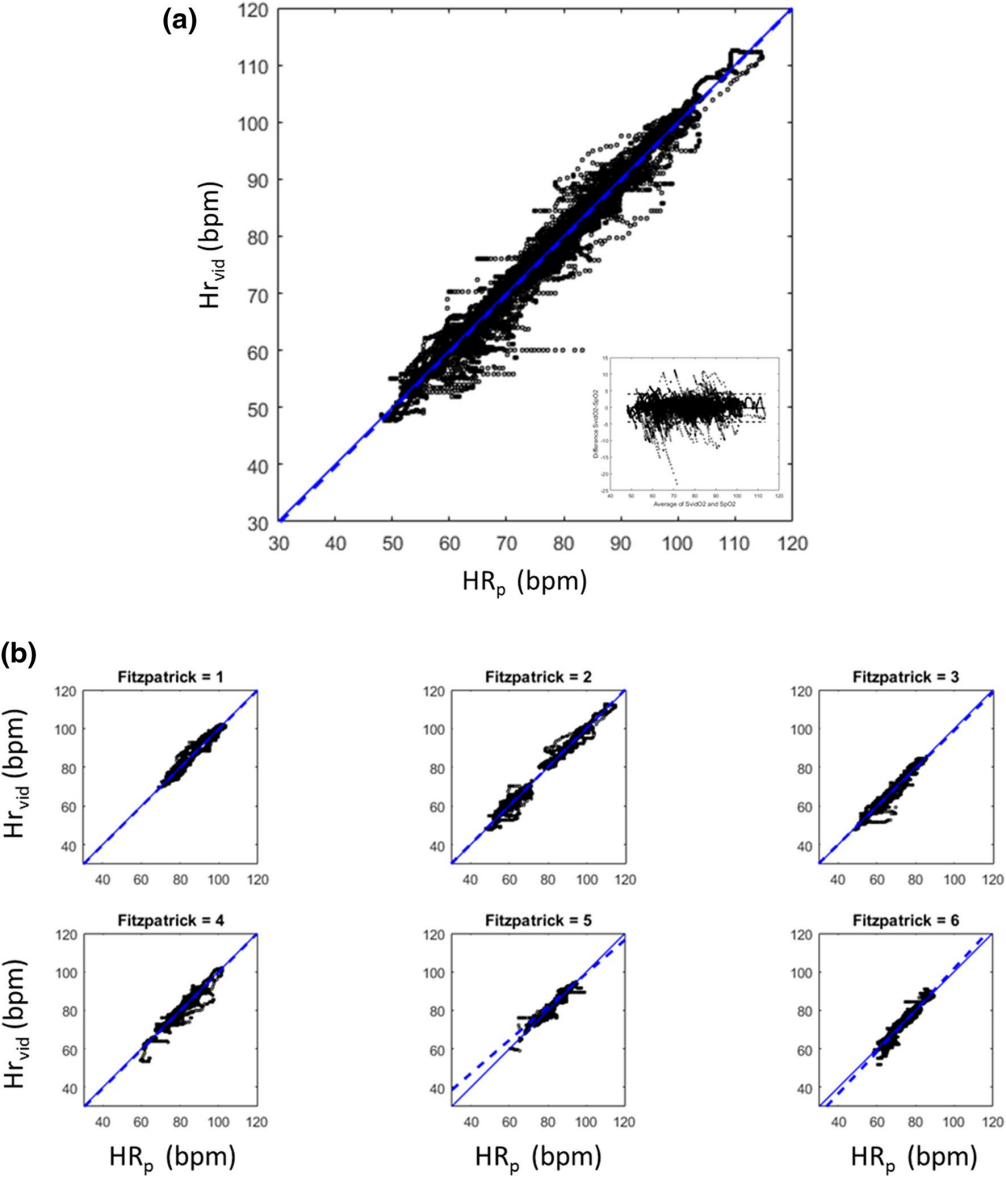
Scatter plots of HRvid against HRp pigmentation. **a** All data. **b** Separated according to Fitzpatrick Skin Pigmentation Scale. Regression line shown as dashed trend line. Expected 1:1 correspondence line shown as continuous thin line. The corresponding Bland–Altman plot is also shown in (**a**)

Figure 5 contains the resulting HR_vid_ time series for all 20 desaturations episodes with the reference HR_p_ signal also plotted for comparison. The variation in heart rate caused by the desaturation events is obvious in the plots. Each subject varied their heart rate over a range (maximum–minimum) induced by the hypoxic challenges. This range of heart rates varied across volunteers from 14.1 to 38.5 bpm, with a mean range of 24.1 bpm. The data is represented more compactly in Fig. 6a which contains the scatter plot of HR_vid_ against HR_p_ for all the data. The figure also includes the associated Bland–Altman analysis plot with the limits of agreement shown. The least squares regression line is plotted on the figure and has the equation: HR_vid_ = 1.00 HR_p_ − 0.39. The Pearson correlation is R = 0.99 (p < 0.0001). The overall bias and RMSD were found to be − 0.21 and 2.15 bpm respectively. The E5, E10 and E25 values were 3.59, 0.35 and 0%. The latter indicating that no computed HR_vid_ deviated by more than 25 bpm from the reference pulse oximeter pulse rate. An uptime of 98.78% was achieved across the whole data set. Figure 6b shows the corresponding scatter plots split according to Fitzpatrick scale, and Table 2 contains the associated individual summary statistics in addition to those of the aggregated data cited above.

**Table 2:** Performance statistics split according to Fitzpatrick Scale Classification

Statistic	Fitzpatrick Scale						
	1	2	3	4	5	6	All
N	2	2	2	2	1	1	10
Bias (bpm)	− 0.26	0.22	− 0.31	− 0.35	− 0.33	− 0.42	− 0.21
RMSD (bpm)	1.74	2.32	1.88	2.04	2.89	2.43	2.15
R	0.98	0.99	0.97	0.97	0.91	0.96	0.99
p value	< 0.01	< 0.01	< 0.01	< 0.01	< 0.01	< 0.01	< 0.01
m (gradient)	1.00	1.00	0.98	1.00	0.93	1.08	1.00
c (intercept) (bpm)	− 0.01	0.05	0.94	− 0.61	5.72	− 6.50	− 0.39
E5 (%)	1.40	4.48	1.95	3.48	6.55	6.72	3.59
E10 (%)	0.00	0.31	0.36	0.52	1.35	0.00	0.35
E25 (%)	0.00	0.00	0.00	0.00	0.00	0.00	0.00
Uptime (%)	99.56	97.71	100.00	99.05	98.54	97.09	98.78

## Discussion

Excellent agreement was found between the computed video heart rates and the reference oximeter pulse rate with an overall RMSD of 2.15 bpm (bias: − 0.21) achieved at a 98.78% uptime. This is within the typical operating ranges of heart rate devices used in practice and well within the accuracy specified in ANSI/AAMI EC13: 2002 where an accuracy of ± 10% or ± 5 bpm, whichever the greater, is specified (ANSI, 2002). RMSDs ranged from 1.74 to 2.89 bpm when split according to skin tone scale, with no obvious pattern discernable Similarly the bias, E5, E10, E25, and uptimes all showed no discernable pattern when split according to skin type. The overall E5 value of 3.59% compares favourably with the work of [22] who found that 17% of heart rates were greater than 5 bpm in their study of camera-based photoplethysmography in critical care patients who were mainly unconscious. Existing ambient room lighting from ceiling-based fluorescent lighting was used in the study. The intensity was measured to be 250 lx at the subject head level. This is a reasonably low intensity room lighting for the clinical setting. Further studies should consider the threshold light intensities below which the technology fails to detect a useful video-PPG. In addition, although light intensity was measured, we did not have equipment available to measure the spectra of the ambient lighting conditions. This is something that will be considered for future studies.

**Fig. 7:**
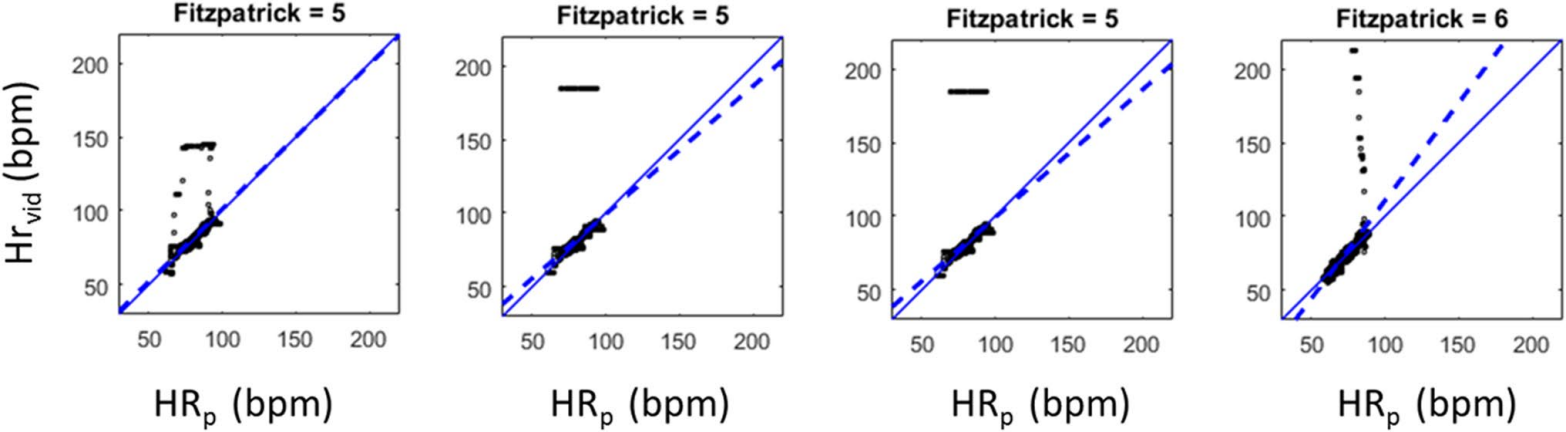
Failure modes associated with other variations of the algorithm. Outliers found in three alternative algorithms for Fitzpatrick Scale V and one for Fitzpatrick Scale VI

Given the small data set (N = 20: 10 subjects each with 2 desaturations) and its use in developing the algorithm, over-training is a possibility, and results must be viewed in this context. Further, this limited data set resulted in only 1 or 2 subjects per Fitzpatrick scale and the results again should be viewed in this context. To this end, the algorithm presented here was compared to four alternative versions containing slight code variations. These were: (1) PPG processed using only the green channel; (2) PPG processed using the red and green channels; (3) A weighted averaging of the FFT spectrum between frames; (4) as (3) but with a longer FFT window of 30 s instead of 18 s. These resulted in reasonably similar performances, but exhibited localised failure modes in short sections of signal. These distinct failure modes for the alternative algorithms are shown in Fig. 7.^1^ Interestingly, three produced noticeable outliers for the Fitzpatrick scale V data and one for the scale VI data; but none failed in this way for both Fitzpatrick scales. In addition to the four variants of the automated algorithm, a semi-manual algorithm was run, whereby the flood field tracking was corrected manually during the processing by changing the tolerances and temporal filtering so that edge flicker on the flood field was minimised. No failure modes such as those of Fig. 7 were found for this manually corrected algorithm version which resulted in an improved overall bias and RMSD of 0.011 and 1.703 bpm respectively. However, these were achieved at a slightly lower uptime of 97.50%. This suggests that improved results may be achievable for a fully automated algorithm, but may be at the expense of marginally poorer uptimes. In lieu of a large dataset with which to construct proper training and test sets for development, the authors find that such consideration of algorithmic variants a useful method for gaining a feel for the likely failure modes of the algorithm when exposed to more data in the field. However, only further data will allow the proper training and testing of the algorithm. Given these caveats regarding the small data set, the algorithm achieves good results for data which covers the range of Fitzpatrick scales and contains a moderate degree of natural motion.

We have described the motion in this study as ‘moderate’. There is, however, no strict definition of motion in this area at present. In fact, the issue of motion has not been rigorously studied to date and many reported studies simply manually remove data where there is motion with no indication of its nature nor criteria given for its removal. In the study presented here we included all signals from all subjects from a real environment in our analysis and thus targeted a 100% uptime; which is a main goal of medical monitoring. Rather than exclude signal, we have attempted to cope with the motion that was present in the study algorithmically.

**Fig. 8:**
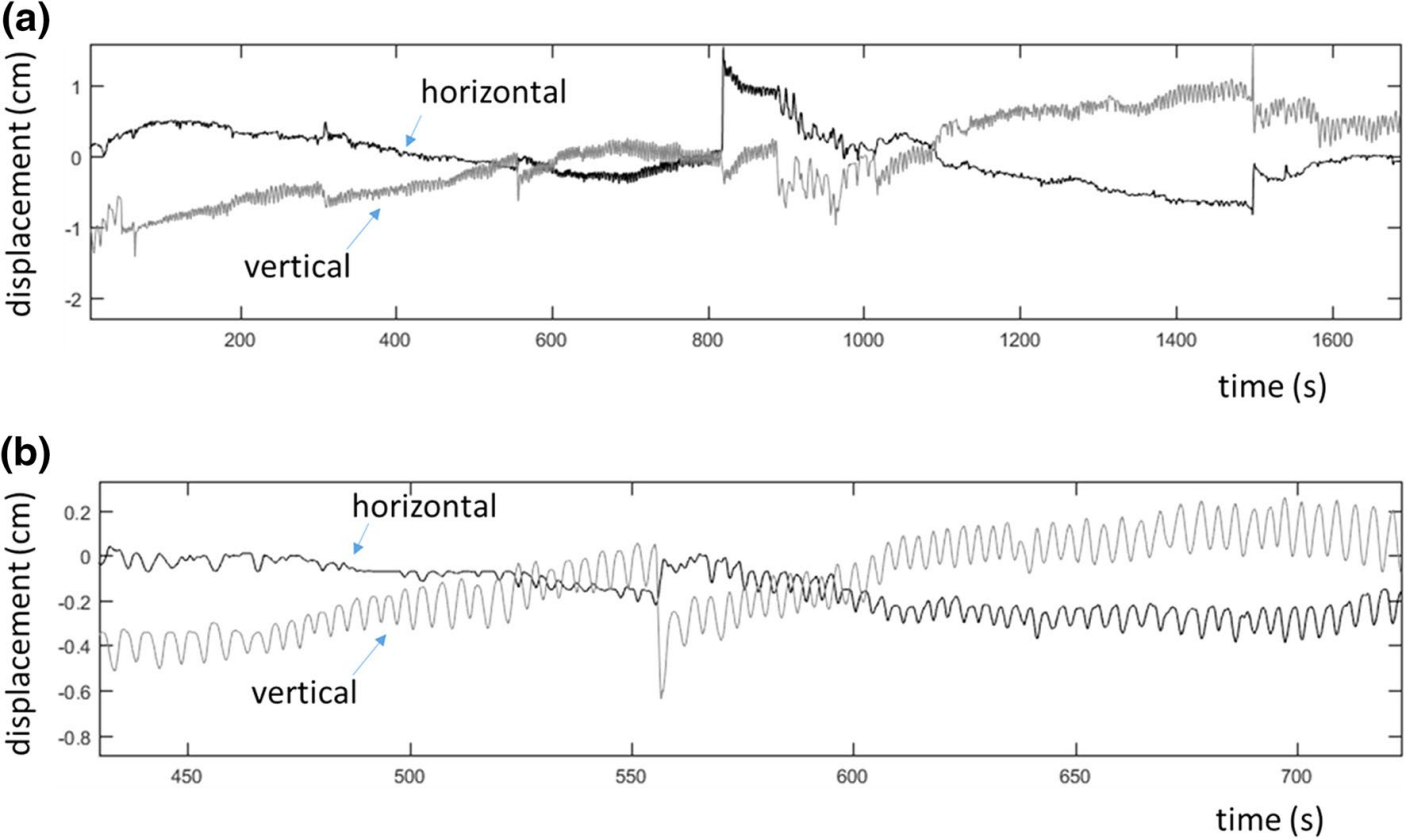
The horizontal and vertical displacement signals. **a** A complete desaturation event. **b** Zoomed in section highlighting respiratory oscillations

Even for traditional pulse oximetry, a mature area of clinical monitoring, there are no universally accepted motion characteristics. Instead, companies develop their own internal protocols and analysis tools for specific studies. Recently, our group proposed a novel motion protocol for video monitoring including yaw, pitch and roll maneuvers of the head [23], although this is at a very early stage and specific to idealized roll, pitch and yaw maneuvers. In fact, most robustness to motion in commercial algorithms for pulse oximetry comes from tuning them using large amounts of patient data from across the spectrum of patient care, as it is very difficult to synthesize the wide variety of complex activities seen in practice. As an example of the motion we experienced in this trial, Fig. 8 contains the horizontal and vertical displacement traces from the centroid of the ROI of one of the desaturation events conducted. A slow drift upwards of the forehead, of around 1.5 cm, may be observed during the 28 min desaturation event. There is a corresponding drift to the right, which is corrected at around 800 s. However, zooming in to the signals reveals the nature of the higher frequency components where a noticeable oscillation at the frequency of respiration can be observed. This is approximately 17.5 breaths per minute (BrPM) at this stage of the desaturation. (This slows to around 13 BrPM towards the end of the study as the subject begins to relax.) The respiratory oscillation has a magnitude of around 0.2 cm in the vertical direction and up to 0.1 cm in the horizontal. The total absolute distance travelled was 1.71 m (as opposed to the displacement over the period of 1.5 cm). Although, seemingly small in magnitude the motion is non-trivial in nature as, without accurate tracking, the constant change of pixel information within a static ROI makes it almost impossible to produce a sensible video-PPG signal for analysis. In addition much of the noise in the signal is within the band of the pulsatile activity, especially during localized head movements by the subjects.

The question, whether video-based heart rate monitoring could replace other modalities for monitoring this vital sign in the clinical environment, is still open. There may, however, be specific clinical use cases where the technology may be adopted more readily in its current state. For example, spot checks by the clinician using, for example, a pulse oximeter may allow intermittent calibration of a camera-based heart rate monitoring system to allow continuous monitoring to take place with reasonable accuracy between checks. The home environment is another area of interest as more monitoring is taking place in order to reduce costs and improve out-of-hospital care. In addition, the monitoring of heart rate using video methods may be combined with other video monitoring technologies relevant to the clinical environment, including the monitoring of oxygen saturation [8, 24], respiratory rate [25], pulse transit times [26], gait, [27] and the detection of falls [28]. Further development of video-based physiological monitoring will require robust algorithms to tackle motion and lighting effects before the technology is mature enough for these application areas.

## Conclusion

The results provide a strong indication of the potential to determine heart rate from video image streams of subjects exhibiting a range of skin pigmentations with moderate motion. Further work is required to fully test this technology in a more rigorous fashion, where a range of motion, skin pigmentations and ambient lighting levels is considered within a comprehensive protocol.

## Footnotes

1 The original 18 s window was chosen from experience. We did not conduct a parametric study because, as we have such limited data, we would be prone to overtraining the algorithm. Typically, a commercial pulse oximeter would have a dynamic window which can be anything in length from around 10 s upwards, and can be relatively long during prolonged noise i.e. > 60 s. In addition, many algorithms use an IIR filter where the new information is added to a weighted average from a sequence of data of increasing length at which point a statistical estimate of the ‘history’ of the utilized data is required, i.e. no fixed window.
